# The chain structure of [Ni(C_4_H_2_O_4_)(C_12_H_8_N_2_)(H_2_O)]_*n*_ with different types of fumarate bridging

**DOI:** 10.1107/S1600536811054614

**Published:** 2012-01-07

**Authors:** Anna Uhrinová, Juraj Kuchár, Juraj Černák

**Affiliations:** a Department of Inorganic Chemistry, Institute of Chemistry, P. J. Šafárik University in Košice, Moyzesova 11, 041 54 Košice, Slovakia

## Abstract

Using modified solvothermal conditions (longer cooling time), beside previously characterized dark-green crystals of [Ni(C_4_H_2_O_4_)(C_12_H_8_N_2_)] (main product), a few light-green crystals of the polymeric title compound, *catena*-poly[[aqua­(1,10-phenanthroline-κ^2^ *N*,*N*′)nickel(II)]-μ-fumarato-κ^2^ *O*:*O*′-[aqua­(1,10-phenanthroline-κ^2^ *N*,*N*′)nickel(II)]-μ-fumarato-κ^4^ *O*,*O*′:*O*′′,*O*′′′], [Ni(C_4_H_2_O_4_)(C_12_H_8_N_2_)(H_2_O)]_*n*_ were isolated. Its crystal structure is made up from zigzag chains, propagating in [001], in which the Ni^2+^ ions are linked alternatively by μ_2_-fumarato and bis-chelating fumarato bridging ligands. The Ni^2+^ ion is coordinated in a deformed octa­hedral geometry by one chelating 1,10-phenanthroline ligand, one aqua ligand in a *cis* position with regard to both *N*-donor atoms and by two different fumarato ligands, each residing with its central C=C bond on an inversion centre, occupying the remaining coordination sites in a *fac* fashion. The chains thus formed are linked by O—H⋯O hydrogen bonds and π–π inter­actions between the aromatic rings of the phenanthroline ligands with a shortest ring centroid separation of 3.4787 (10) Å.

## Related literature

For Ni^2+^ complexes containing both fum and phen ligands (fum = fumarato, phen = 1,10-phenanthroline), see: Černák *et al.* (2009 ▶) for [Ni(fum)(phen)] with a two-dimensional structure and Ma *et al.* (2003 ▶) for [Ni_2_(phen)_4_(fum)(H_2_O)_2_]fum·16H_2_O with an ionic structure containing a dinuclear complex cation. For an Ni^2+^ complex, [Ni_2_(fum)_2_(py)_6_]·2py (py = pyridine), exhibiting a one-dimensional structure with the same type of fumarato bridging ligands, see: Mori *et al.* (2004 ▶); Marsh *et al.* (2005 ▶).
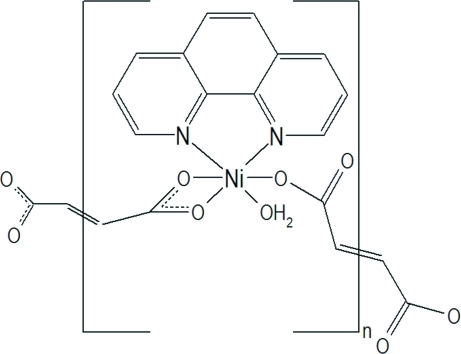


## Experimental

### 

#### Crystal data

[Ni(C_4_H_2_O_4_)(C_12_H_8_N_2_)(H_2_O)]
*M* *_r_* = 370.99
Triclinic, 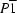

*a* = 7.8998 (4) Å
*b* = 9.8238 (5) Å
*c* = 11.3815 (8) Å
α = 68.677 (6)°
β = 70.141 (6)°
γ = 89.655 (5)°
*V* = 766.89 (8) Å^3^
*Z* = 2
Mo *K*α radiation
μ = 1.29 mm^−1^
*T* = 173 K
0.46 × 0.27 × 0.12 mm

#### Data collection

Oxford Diffraction Xcalibur Sapphire2 diffractometer
Absorption correction: analytical [Clark & Reid (1995 ▶) in *CrysAlis PRO* (Oxford Diffraction, 2009 ▶)] *T*_min_ = 0.658, *T*_max_ = 0.866
12619 measured reflections
3173 independent reflections
2972 reflections with *I* > 2σ(*I*)
*R*_int_ = 0.022

#### Refinement

*R*[*F* ^2^ > 2σ(*F* ^2^)] = 0.022
*wR*(*F* ^2^) = 0.057
*S* = 1.04
3173 reflections
217 parameters
H-atom parameters constrained
Δρ_max_ = 0.33 e Å^−3^
Δρ_min_ = −0.25 e Å^−3^

### 

Data collection: *CrysAlis PRO* (Oxford Diffraction, 2009 ▶); cell refinement: *CrysAlis PRO*; data reduction: *CrysAlis PRO*; program(s) used to solve structure: *SHELXS97* (Sheldrick, 2008 ▶); program(s) used to refine structure: *SHELXL97* (Sheldrick, 2008 ▶); molecular graphics: *DIAMOND* (Crystal Impact, 2009 ▶); software used to prepare material for publication: *SHELXL97*.

## Supplementary Material

Crystal structure: contains datablock(s) I, global. DOI: 10.1107/S1600536811054614/wm2574sup1.cif

Structure factors: contains datablock(s) I. DOI: 10.1107/S1600536811054614/wm2574Isup2.hkl

Additional supplementary materials:  crystallographic information; 3D view; checkCIF report

## Figures and Tables

**Table 1:** Hydrogen-bond geometry (Å, °)

*D*—H⋯*A*	*D*—H	H⋯*A*	*D*⋯*A*	*D*—H⋯*A*
O1—H1O1⋯O5	0.85	1.78	2.6085 (15)	163
O1—H2O1⋯O2^i^	0.85	1.93	2.7820 (15)	177

Symmetry code: (i) 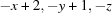.

**Fig. 1.**
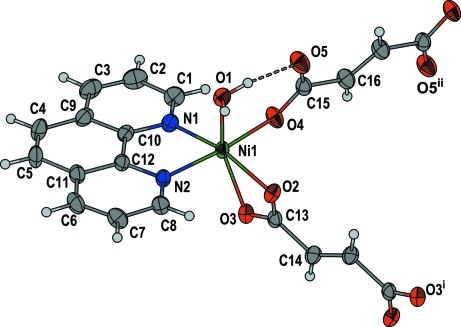
Coordination environment around the Ni2+ ion in (I). Displacement ellipsoids are drawn at the 50% probability level. [Symmetry codes: i) 1 - x, 1 - y, -z; ii) 1 - x, 1 - y, 1 - z.]

**Fig. 2.**
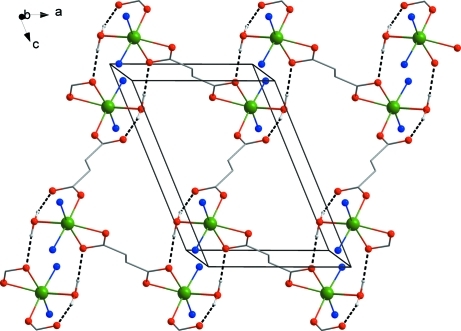
View of the hydrogen bonding system in (I) linking neighbouring chains. Colour code Ni: green, O: red, N: blue, H: white spheres. Hydrogen atoms of the fumarato ligands are omitted as well as the phen ligands but their N donor atoms for clarity.

**Fig. 3.**
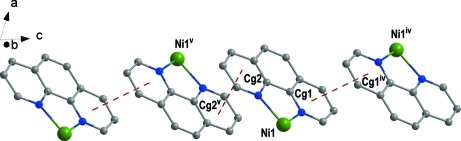
View of the π—π interactions between the phen ligands in (I). [Symmetry codes: iv) 2 - x, -y, 1 - z; v) 2 - x, -y, -z.]

## supplementary crystallographic information

### Comment

Within our broader study on low-dimensional compounds of Ni(II) in the aqueous-alcoholic system Ni(II)-fum-phen (fum = fumarato, phen = 1,10-phenanthroline) using solvothermal conditions, we have isolated the title compound [Ni(fum)(H_2_O)(phen)] (I) in the form of few light-green crystals accompanying the major dark-green crystalline product [Ni(fum)(phen)] (II) (Černák et al., 2009). Such dark-green crystals were only formed if the reaction mixture was cooled from 373 K down to room temperature during 8 h. When the cooling time was elongated to 20 h, some light-green crystals of (I) likewise appeared.

The crystal structure of (I) is built up of zigzag chains exhibiting the backbone composition [–Ni–fum–Ni–fum–]_n_ propagating parallel to the [001] direction. Within the chain the Ni^2+^ ions are linked alternatively by µ_2_-fumarato and bis-chelating fumarato bridging ligands (Fig. 1, Fig. 2). The same type of chains is observed in [Ni_2_(fum)_2_(py)_6_]^.^2py (Mori et al., 2004; Marsh et al., 2005). On the other hand, the dark-green [Ni(fum)(phen)] exhibits a two-dimensional crystal structure built up of dimers of crystallographically non-equivalent Ni^2+^ ions linked by two different types of bridging fumarato ligands (Černák et al., 2009).

The Ni^2+^ central atom in (I) is octahedrally coordinated in a deformed NiN_2_O_3_O donor set by one chelating phen ligand, one aqua ligand placed in cis-position with regard to both donor nitrogen atoms of the phen ligand, and three coordination sites are occupied by oxygen atoms originating from two chemically different fum ligands, one coordinating through one O-donor atom, and the second one coordinating in a chelating fashion (Fig. 2). The presence of an additional aqua ligand in the coordination sphere of the Ni(II) atom lowers the number of free coordination sites available for polymerization from four in (II) to three in (I). Consequently, a crystal structure with lower dimensionality is realised.

The mean value of the Ni–N bond lengths (2.07 (2) Å) is close to that observed in [Ni_2_(phen)_4_(fum)(H_2_O)_2_]fum^.^16H_2_O (2.096 (1) Å) (Ma et al., 2003). In (I) two crystallographically independent fum^2-^ ligands in the asymmetric unit reside on an inversion centre. The chelating fum ligand coordinates in an unsymmetrical fashion and the corresponding Ni—O bonds are longer with respect to the Ni—O bond of the monodentately coordinating fum ligand (Table 2). A similar situation as to the Ni–O bond lengths was observed in [Ni_2_(fum)_2_(py)_6_]^.^2py (Mori et al., 2004). The Ni—O bond length of the aqua ligand exhibits an usual value (Ma et al., 2003).

Both hydrogen atoms of the aqua ligand are involved in rather strong hydrogen bonds of the O—H···O type (Fig. 3, Table 3). The H1O1 atom forms an intermolecular hydrogen bond with the not coordinating O5 atom from the carboxylate group. On the other hand, the H2O1 atom participates in an intermolecular hydrogen bond with the O2 atom (symmetry code 2 - x, 1 - y, -z) linking neighbouring chains; due to symmetry operators (centre of symmetry) the chains are linked with a pair of such hydrogen bond forming a ring arrangement R_2_^2^(8).

Moreover, the chains interact also through π—π interactions between the aromatic rings of the phen ligands (Fig. 3). The distances between the centres of gravity, Cgi of the aromatic rings, are: 3.7942 (11) Å for Cg1—Cg1^iv^ (Cg1 is the centre of gravity of the aromatic ring formed by atoms N1, C10, C9, C3, C2, C1) and 3.4787 (10) Å for Cg2···Cg2^v^ (Cg2 is the centre of gravity of the aromatic ring formed by atoms N2, C8, C7, C6, C11, C12). Similar π—π interactions were also observed in the structure of the dark-green [Ni(fum)(phen)] (Černák et al., 2009).

### Experimental

A Parr reaction vessel (total volume 20 cm^3^) was filled with 6 cm^3^ of aqueous-ethanolic solution (1:1) containing 238 mg (1 mmol) of NiCl_2_^.^6H_2_O, 116 mg (1 mmol) of fumaric acid and 396 mg (2 mmol) of 1,10-phenantroline. Finally, 112 mg (2 mmol) of solid KOH was added. The closed reaction vessel was heated by uniform heating rate to 373 K during one hour and then was left at this temperature for 80 h. The reaction vessel was then uniformly cooled to room temperature during 20 h. The product consisted of dark-green crystals of [Ni(fum)(phen)] (main product) and few light-green crystals of [Ni(fum)(H_2_O)(phen)]. The mixture of crystals was separated from the mother liquor by filtration and dried at laboratory temperature. The light-green crystals were picked up and used for X-ray study.

### Refinement

The hydrogen atoms from the water molecule were located in a difference map, but their positions were constrained by geometric parameters to values of 0.850 Å for the O—H bond. The isotropic displacement parameters of the hydrogen atoms were tied to those of the parent oxygen atoms with *U*_iso_(H) = 1.5Ueq(O). The hydrogen atoms of the phenantroline ligand and fumarato(2-) ligand were positioned geometrically with C—H = 0.950 Å and constrained to ride on their parent atoms with *U*_iso_(H) = 1.2Ueq(C).

### Crystal data

**Table:**

[Ni(C_4_H_2_O_4_)(C_12_H_8_N_2_)(H_2_O)]	*Z* = 2
*M**_r_* = 370.99	*F*(000) = 380
Triclinic, *P*1	*D*_x_ = 1.607 Mg m^−^^3^
Hall symbol: -P 1	Mo *K*α radiation, λ = 0.71073 Å
*a* = 7.8998 (4) Å	Cell parameters from 9394 reflections
*b* = 9.8238 (5) Å	θ = 3.3–29.4°
*c* = 11.3815 (8) Å	µ = 1.29 mm^−^^1^
α = 68.677 (6)°	*T* = 173 K
β = 70.141 (6)°	Prism, light-green
γ = 89.655 (5)°	0.46 × 0.27 × 0.12 mm
*V* = 766.89 (8) Å^3^	

### Data collection

**Table:**

Oxford Diffraction Xcalibur Sapphire2 diffractometer	3173 independent reflections
Radiation source: Enhance (Mo) X-ray Source	2972 reflections with *I* > 2σ(*I*)
graphite	*R*_int_ = 0.022
Detector resolution: 8.3438 pixels mm^-1^	θ_max_ = 26.5°, θ_min_ = 3.3°
ω scans	*h* = −9→9
Absorption correction: analytical [Clark & Reid (1995) in *CrysAlis PRO* (Oxford Diffraction, 2009)]	*k* = −12→12
*T*_min_ = 0.658, *T*_max_ = 0.866	*l* = −14→14
12619 measured reflections	

### Refinement

**Table:**

Refinement on *F*^2^	Primary atom site location: structure-invariant direct methods
Least-squares matrix: full	Secondary atom site location: difference Fourier map
*R*[*F*^2^ > 2σ(*F*^2^)] = 0.022	Hydrogen site location: inferred from neighbouring sites
*wR*(*F*^2^) = 0.057	H-atom parameters constrained
*S* = 1.04	*w* = 1/[σ^2^(*F*_o_^2^) + (0.0268*P*)^2^ + 0.3218*P*] where *P* = (*F*_o_^2^ + 2*F*_c_^2^)/3
3173 reflections	(Δ/σ)_max_ = 0.001
217 parameters	Δρ_max_ = 0.33 e Å^−^^3^
0 restraints	Δρ_min_ = −0.24 e Å^−^^3^

### Special details

**Table:**

Geometry. All e.s.d.'s (except the e.s.d. in the dihedral angle between two l.s. planes) are estimated using the full covariance matrix. The cell e.s.d.'s are taken into account individually in the estimation of e.s.d.'s in distances, angles and torsion angles; correlations between e.s.d.'s in cell parameters are only used when they are defined by crystal symmetry. An approximate (isotropic) treatment of cell e.s.d.'s is used for estimating e.s.d.'s involving l.s. planes.
Refinement. Refinement of *F*^2^ against ALL reflections. The weighted *R*-factor *wR* and goodness of fit *S* are based on *F*^2^, conventional *R*-factors *R* are based on *F*, with *F* set to zero for negative *F*^2^. The threshold expression of *F*^2^ > σ(*F*^2^) is used only for calculating *R*-factors(gt) *etc*. and is not relevant to the choice of reflections for refinement. *R*-factors based on *F*^2^ are statistically about twice as large as those based on *F*, and *R*- factors based on ALL data will be even larger.

### Fractional atomic coordinates and isotropic or equivalent isotropic displacement parameters (Å^2^)

**Table:**

	*x*	*y*	*z*	*U*_iso_*/*U*_eq_	
Ni1	0.83574 (2)	0.255869 (19)	0.208342 (18)	0.02046 (7)	
O1	1.05052 (14)	0.37310 (12)	0.20856 (11)	0.0254 (2)	
H1O1	0.9946	0.4226	0.2539	0.038*	
H2O1	1.1060	0.4310	0.1268	0.038*	
O2	0.77961 (14)	0.43278 (11)	0.05893 (11)	0.0246 (2)	
O3	0.60066 (15)	0.22129 (11)	0.15882 (11)	0.0256 (2)	
O4	0.65578 (15)	0.30773 (12)	0.35633 (11)	0.0297 (2)	
O5	0.82308 (16)	0.48624 (13)	0.36300 (12)	0.0351 (3)	
N1	0.85113 (17)	0.05252 (14)	0.34101 (12)	0.0240 (3)	
N2	1.02443 (17)	0.18287 (13)	0.07328 (12)	0.0215 (3)	
C1	0.7599 (2)	−0.01127 (18)	0.47314 (16)	0.0311 (4)	
H1	0.6707	0.0385	0.5153	0.037*	
C2	0.7899 (3)	−0.1492 (2)	0.55307 (17)	0.0373 (4)	
H2	0.7221	−0.1916	0.6476	0.045*	
C3	0.9174 (3)	−0.22201 (19)	0.49376 (18)	0.0370 (4)	
H3	0.9389	−0.3155	0.5469	0.044*	
C4	1.1536 (3)	−0.22521 (19)	0.28194 (19)	0.0375 (4)	
H4	1.1810	−0.3187	0.3300	0.045*	
C5	1.2444 (2)	−0.15787 (19)	0.14727 (19)	0.0351 (4)	
H5	1.3352	−0.2046	0.1026	0.042*	
C6	1.2939 (2)	0.05748 (19)	−0.07064 (18)	0.0321 (4)	
H6	1.3850	0.0156	−0.1209	0.039*	
C7	1.2463 (2)	0.19111 (18)	−0.13455 (16)	0.0311 (4)	
H7	1.3051	0.2431	−0.2294	0.037*	
C8	1.1105 (2)	0.25035 (17)	−0.05917 (15)	0.0263 (3)	
H8	1.0786	0.3429	−0.1048	0.032*	
C9	1.0168 (2)	−0.15825 (17)	0.35373 (17)	0.0291 (3)	
C10	0.9774 (2)	−0.01967 (16)	0.28145 (15)	0.0228 (3)	
C11	1.2066 (2)	−0.01704 (17)	0.07015 (17)	0.0271 (3)	
C12	1.0727 (2)	0.05085 (16)	0.13778 (15)	0.0221 (3)	
C13	0.6336 (2)	0.35570 (16)	0.08395 (14)	0.0219 (3)	
C14	0.4973 (2)	0.42762 (17)	0.02642 (16)	0.0249 (3)	
H14	0.4064	0.3645	0.0340	0.030*	
C15	0.6762 (2)	0.41233 (16)	0.39247 (15)	0.0247 (3)	
C16	0.5037 (2)	0.44635 (18)	0.47800 (16)	0.0280 (3)	
H16	0.4000	0.3893	0.4987	0.034*	

### Atomic displacement parameters (Å^2^)

**Table:**

	*U*^11^	*U*^22^	*U*^33^	*U*^12^	*U*^13^	*U*^23^
Ni1	0.01891 (11)	0.02183 (11)	0.01896 (10)	0.00744 (7)	−0.00459 (8)	−0.00816 (8)
O1	0.0204 (6)	0.0307 (6)	0.0219 (5)	0.0058 (4)	−0.0051 (4)	−0.0091 (4)
O2	0.0189 (6)	0.0254 (5)	0.0260 (5)	0.0056 (4)	−0.0082 (5)	−0.0062 (4)
O3	0.0252 (6)	0.0218 (5)	0.0281 (6)	0.0065 (4)	−0.0082 (5)	−0.0093 (4)
O4	0.0258 (6)	0.0322 (6)	0.0296 (6)	0.0048 (5)	−0.0008 (5)	−0.0186 (5)
O5	0.0254 (6)	0.0413 (7)	0.0394 (7)	0.0038 (5)	−0.0029 (5)	−0.0247 (6)
N1	0.0222 (7)	0.0256 (6)	0.0207 (6)	0.0043 (5)	−0.0059 (5)	−0.0068 (5)
N2	0.0209 (7)	0.0216 (6)	0.0208 (6)	0.0059 (5)	−0.0059 (5)	−0.0082 (5)
C1	0.0282 (9)	0.0358 (9)	0.0236 (8)	0.0030 (7)	−0.0054 (7)	−0.0088 (7)
C2	0.0402 (11)	0.0378 (9)	0.0226 (8)	−0.0031 (8)	−0.0091 (8)	−0.0011 (7)
C3	0.0461 (11)	0.0285 (8)	0.0326 (9)	0.0039 (8)	−0.0212 (9)	−0.0012 (7)
C4	0.0444 (11)	0.0270 (8)	0.0482 (11)	0.0179 (8)	−0.0262 (9)	−0.0139 (8)
C5	0.0329 (10)	0.0330 (9)	0.0480 (10)	0.0185 (7)	−0.0175 (8)	−0.0226 (8)
C6	0.0255 (9)	0.0365 (9)	0.0368 (9)	0.0070 (7)	−0.0043 (7)	−0.0233 (8)
C7	0.0297 (9)	0.0340 (9)	0.0235 (8)	0.0004 (7)	−0.0006 (7)	−0.0126 (7)
C8	0.0288 (9)	0.0247 (7)	0.0224 (7)	0.0040 (6)	−0.0069 (7)	−0.0079 (6)
C9	0.0323 (9)	0.0249 (7)	0.0327 (8)	0.0061 (7)	−0.0182 (7)	−0.0078 (7)
C10	0.0219 (8)	0.0222 (7)	0.0249 (7)	0.0043 (6)	−0.0104 (6)	−0.0080 (6)
C11	0.0233 (8)	0.0280 (8)	0.0348 (8)	0.0077 (6)	−0.0108 (7)	−0.0172 (7)
C12	0.0201 (8)	0.0226 (7)	0.0257 (7)	0.0049 (6)	−0.0090 (6)	−0.0111 (6)
C13	0.0202 (8)	0.0246 (7)	0.0204 (7)	0.0080 (6)	−0.0047 (6)	−0.0106 (6)
C14	0.0177 (8)	0.0291 (7)	0.0283 (8)	0.0061 (6)	−0.0079 (6)	−0.0119 (6)
C15	0.0263 (8)	0.0258 (7)	0.0193 (7)	0.0080 (6)	−0.0049 (6)	−0.0090 (6)
C16	0.0223 (8)	0.0322 (8)	0.0260 (8)	0.0036 (6)	−0.0016 (7)	−0.0140 (7)

### Geometric parameters (Å, °)

**Table:**

Ni1—O4	2.0263 (10)	C3—H3	0.9500
Ni1—N1	2.0527 (12)	C4—C5	1.350 (3)
Ni1—O1	2.0576 (11)	C4—C9	1.432 (2)
Ni1—N2	2.0811 (12)	C4—H4	0.9500
Ni1—O2	2.1124 (10)	C5—C11	1.436 (2)
Ni1—O3	2.1771 (11)	C5—H5	0.9500
O1—H1O1	0.8500	C6—C7	1.371 (2)
O1—H2O1	0.8500	C6—C11	1.408 (2)
O2—C13	1.2711 (18)	C6—H6	0.9500
O3—C13	1.2540 (18)	C7—C8	1.398 (2)
O4—C15	1.2671 (18)	C7—H7	0.9500
O5—C15	1.2470 (19)	C8—H8	0.9500
N1—C1	1.326 (2)	C9—C10	1.408 (2)
N1—C10	1.3563 (19)	C10—C12	1.438 (2)
N2—C8	1.3253 (19)	C11—C12	1.403 (2)
N2—C12	1.3631 (19)	C13—C14	1.490 (2)
C1—C2	1.402 (2)	C14—C14^i^	1.322 (3)
C1—H1	0.9500	C14—H14	0.9127
C2—C3	1.365 (3)	C15—C16	1.498 (2)
C2—H2	0.9500	C16—C16^ii^	1.315 (3)
C3—C9	1.407 (2)	C16—H16	0.9056
			
O4—Ni1—N1	93.22 (5)	C5—C4—C9	121.22 (15)
O4—Ni1—O1	92.07 (4)	C5—C4—H4	119.4
N1—Ni1—O1	98.29 (5)	C9—C4—H4	119.4
O4—Ni1—N2	173.69 (5)	C4—C5—C11	121.40 (15)
N1—Ni1—N2	80.59 (5)	C4—C5—H5	119.3
O1—Ni1—N2	87.68 (5)	C11—C5—H5	119.3
O4—Ni1—O2	90.64 (4)	C7—C6—C11	119.38 (14)
N1—Ni1—O2	165.34 (5)	C7—C6—H6	120.3
O1—Ni1—O2	95.70 (4)	C11—C6—H6	120.3
N2—Ni1—O2	95.66 (4)	C6—C7—C8	119.52 (15)
O4—Ni1—O3	84.64 (4)	C6—C7—H7	120.2
N1—Ni1—O3	104.64 (5)	C8—C7—H7	120.2
O1—Ni1—O3	156.97 (4)	N2—C8—C7	122.79 (14)
N2—Ni1—O3	98.02 (4)	N2—C8—H8	118.6
O2—Ni1—O3	61.64 (4)	C7—C8—H8	118.6
O4—Ni1—C13	84.46 (5)	C3—C9—C10	116.94 (15)
N1—Ni1—C13	135.31 (5)	C3—C9—C4	124.20 (15)
O1—Ni1—C13	126.36 (5)	C10—C9—C4	118.86 (15)
N2—Ni1—C13	100.76 (5)	N1—C10—C9	122.99 (14)
O2—Ni1—C13	31.19 (4)	N1—C10—C12	117.23 (13)
O3—Ni1—C13	30.68 (4)	C9—C10—C12	119.78 (14)
Ni1—O1—H1O1	100.8	C12—C11—C6	117.24 (14)
Ni1—O1—H2O1	106.0	C12—C11—C5	118.60 (15)
H1O1—O1—H2O1	109.3	C6—C11—C5	124.15 (15)
C13—O2—Ni1	89.42 (8)	N2—C12—C11	123.04 (14)
C13—O3—Ni1	86.97 (9)	N2—C12—C10	116.83 (13)
C15—O4—Ni1	127.93 (10)	C11—C12—C10	120.13 (13)
C1—N1—C10	118.30 (13)	O3—C13—O2	121.07 (13)
C1—N1—Ni1	128.64 (11)	O3—C13—C14	119.63 (13)
C10—N1—Ni1	113.02 (10)	O2—C13—C14	119.28 (13)
C8—N2—C12	118.02 (13)	O3—C13—Ni1	62.35 (8)
C8—N2—Ni1	129.72 (10)	O2—C13—Ni1	59.39 (7)
C12—N2—Ni1	112.00 (10)	C14—C13—Ni1	170.01 (10)
N1—C1—C2	122.60 (16)	C14^i^—C14—C13	122.62 (18)
N1—C1—H1	118.7	C14^i^—C14—H14	122.2
C2—C1—H1	118.7	C13—C14—H14	115.2
C3—C2—C1	119.33 (16)	O5—C15—O4	126.05 (14)
C3—C2—H2	120.3	O5—C15—C16	119.26 (13)
C1—C2—H2	120.3	O4—C15—C16	114.68 (14)
C2—C3—C9	119.83 (15)	C16^ii^—C16—C15	123.7 (2)
C2—C3—H3	120.1	C16^ii^—C16—H16	119.5
C9—C3—H3	120.1	C15—C16—H16	116.8
			
O4—Ni1—O2—C13	−78.18 (8)	C2—C3—C9—C4	179.82 (16)
N1—Ni1—O2—C13	27.1 (2)	C5—C4—C9—C3	−179.90 (17)
O1—Ni1—O2—C13	−170.32 (8)	C5—C4—C9—C10	0.0 (2)
N2—Ni1—O2—C13	101.46 (8)	C1—N1—C10—C9	−0.6 (2)
O3—Ni1—O2—C13	5.38 (8)	Ni1—N1—C10—C9	177.36 (12)
O4—Ni1—O3—C13	88.17 (9)	C1—N1—C10—C12	179.14 (14)
N1—Ni1—O3—C13	−179.90 (8)	Ni1—N1—C10—C12	−2.93 (16)
O1—Ni1—O3—C13	5.53 (15)	C3—C9—C10—N1	0.4 (2)
N2—Ni1—O3—C13	−97.59 (9)	C4—C9—C10—N1	−179.51 (14)
O2—Ni1—O3—C13	−5.46 (8)	C3—C9—C10—C12	−179.28 (14)
N1—Ni1—O4—C15	117.90 (13)	C4—C9—C10—C12	0.8 (2)
O1—Ni1—O4—C15	19.47 (13)	C7—C6—C11—C12	0.4 (2)
O2—Ni1—O4—C15	−76.25 (13)	C7—C6—C11—C5	179.43 (15)
O3—Ni1—O4—C15	−137.69 (13)	C4—C5—C11—C12	0.3 (2)
C13—Ni1—O4—C15	−106.87 (13)	C4—C5—C11—C6	−178.73 (17)
O4—Ni1—N1—C1	3.18 (14)	C8—N2—C12—C11	−0.9 (2)
O1—Ni1—N1—C1	95.75 (14)	Ni1—N2—C12—C11	−175.67 (11)
N2—Ni1—N1—C1	−178.02 (14)	C8—N2—C12—C10	179.84 (13)
O2—Ni1—N1—C1	−101.8 (2)	Ni1—N2—C12—C10	5.10 (16)
O3—Ni1—N1—C1	−82.11 (14)	C6—C11—C12—N2	0.4 (2)
C13—Ni1—N1—C1	−82.18 (15)	C5—C11—C12—N2	−178.66 (14)
O4—Ni1—N1—C10	−174.48 (10)	C6—C11—C12—C10	179.62 (14)
O1—Ni1—N1—C10	−81.92 (10)	C5—C11—C12—C10	0.6 (2)
N2—Ni1—N1—C10	4.31 (10)	N1—C10—C12—N2	−1.5 (2)
O2—Ni1—N1—C10	80.5 (2)	C9—C10—C12—N2	178.18 (13)
O3—Ni1—N1—C10	100.22 (10)	N1—C10—C12—C11	179.20 (13)
C13—Ni1—N1—C10	100.15 (11)	C9—C10—C12—C11	−1.1 (2)
N1—Ni1—N2—C8	−179.04 (14)	Ni1—O3—C13—O2	9.36 (13)
O1—Ni1—N2—C8	−80.24 (14)	Ni1—O3—C13—C14	−168.84 (12)
O2—Ni1—N2—C8	15.26 (14)	Ni1—O2—C13—O3	−9.63 (14)
O3—Ni1—N2—C8	77.35 (14)	Ni1—O2—C13—C14	168.57 (12)
C13—Ni1—N2—C8	46.36 (14)	O4—Ni1—C13—O3	−88.83 (8)
N1—Ni1—N2—C12	−5.08 (10)	N1—Ni1—C13—O3	0.14 (11)
O1—Ni1—N2—C12	93.73 (10)	O1—Ni1—C13—O3	−177.32 (7)
O2—Ni1—N2—C12	−170.78 (10)	N2—Ni1—C13—O3	87.59 (9)
O3—Ni1—N2—C12	−108.69 (10)	O2—Ni1—C13—O3	170.69 (13)
C13—Ni1—N2—C12	−139.67 (10)	O4—Ni1—C13—O2	100.48 (8)
C10—N1—C1—C2	0.4 (2)	N1—Ni1—C13—O2	−170.55 (8)
Ni1—N1—C1—C2	−177.15 (12)	O1—Ni1—C13—O2	11.99 (10)
N1—C1—C2—C3	−0.1 (3)	N2—Ni1—C13—O2	−83.10 (8)
C1—C2—C3—C9	0.0 (3)	O3—Ni1—C13—O2	−170.69 (13)
C9—C4—C5—C11	−0.6 (3)	O3—C13—C14—C14^i^	164.10 (18)
C11—C6—C7—C8	−0.7 (2)	O2—C13—C14—C14^i^	−14.1 (3)
C12—N2—C8—C7	0.6 (2)	Ni1—O4—C15—O5	−15.4 (2)
Ni1—N2—C8—C7	174.28 (11)	Ni1—O4—C15—C16	164.36 (10)
C6—C7—C8—N2	0.2 (2)	O5—C15—C16—C16^ii^	1.9 (3)
C2—C3—C9—C10	−0.1 (2)	O4—C15—C16—C16^ii^	−177.9 (2)

Symmetry codes: (i) −*x*+1, −*y*+1, −*z*; (ii) −*x*+1, −*y*+1, −*z*+1.

### Hydrogen-bond geometry (Å, °)

**Table:**

*D*—H···*A*	*D*—H	H···*A*	*D*···*A*	*D*—H···*A*
O1—H1O1···O5	0.85	1.78	2.6085 (15)	163
O1—H2O1···O2^iii^	0.85	1.93	2.7820 (15)	177

Symmetry codes: (iii) −*x*+2, −*y*+1, −*z*.
